# Mortality pattern trends and disparities among Chinese from 2004 to 2016

**DOI:** 10.1186/s12889-019-7163-9

**Published:** 2019-09-02

**Authors:** Jicun Zhu, Lingling Cui, Kehui Wang, Chen Xie, Nan Sun, Fei Xu, Qixin Tang, Changqing Sun

**Affiliations:** 1 College of Public Health, Zhengzhou University, 100 Science Avenue, Zhengzhou, 450001 Henan People’s Republic of China; 2 The First Affiliated Hospital of Henan University of Traditional Chinese Medicine, People’s Road, Zhengzhou, 450000 Henan People’s Republic of China; 3 Department of Management Information Systems, University of Georgia Terry College of Business Athens, Georgia, 30602 USA

**Keywords:** Morbidity and mortality trends, Cause of death, Health disparities, Non-communicable diseases, Burden of disease

## Abstract

**Background:**

With the changes in environmental, medical technique, population structure and national health projects, human mortality rates have undergone great changes all over the world. According to “World Health Statistics 2016: Monitoring Health for the SDGs (Sustainable Development Goals)”, we can draw a globally vision about life expectancy and cause of death; also, significant inequality still persists within and among countries. This study was designed to research into the trend of mortality pattern in China, evaluate the disparities of age-specific and disease-specific mortality rates between male and female, and provides a scientific basis for further prevention strategies and policies design.

**Methods:**

Data from the Chinese Disease Surveillance Points system were used to calculate crude and age-adjusted death rates, annual percent changes (APC) for men and women during 2004 to 2016. Age-standardized mortality rates (ASMR) were performed through the direct method with the World Health Organization’s World Standard Population. APC, according to log linear model, was adopted to describe the mortality rate trend. The *χ*^*2*^ test was used to compare differences between age-specific and cause-specific mortality rates of men and women. Data analysis and figures were completed by R software.

**Results:**

The mortality rates of men and women have decreased significantly (*P* < 0.05) during 2004–2016, and the APC were1.98 and 2.45%, respectively. In 2016, the crude mortality rate (CMR) and ASMR in all causes of death were 658.50 and 490.28 per 100,000 per year, respectively. The 5 leading causes of death were malignant neoplasm, cerebrovascular disease, heart disease, COPD, and accidental injury. The mortality rates of men were higher than that of women in all age groups.

**Conclusions:**

There are severe health gaps and disparities between male and female, and the chronic non-communicable diseases continue to be a serious health threat to Chinese residents.

**Electronic supplementary material:**

The online version of this article (10.1186/s12889-019-7163-9) contains supplementary material, which is available to authorized users.

## Background

The analysis on life expectancy and causes of death is the primary project to reflect the health level of residents, which is helpful to evaluate the effectiveness of disease prevention and control, and to determine the next step in public health work. With the rapid growth of economy since 1979, mortality rates of infants and infectious diseases have declined sharply and the health status of Chinese people has been improved dramatically. The Human Development Index (HDI) is an important metric, representing the basic components of human well-being, which integrates health, education and income. According to the United Nations Development Programme (UNDP) report, in 2015, life expectancy in China reached 76.00 years and HDI reached 0.74 [1]. However, as the changes of environment, medical technique, population structure and national health policies, the disease-specific mortality rates have undergone great changes in China. In 2016, World Health Organization (WHO) researched on global cause-of-death patterns, and stated that non-communicable diseases (NCDs) kill 40 million people each year, equivalent to 70% of all deaths globally [2]. Moreover, significant differences persist among countries, nearly 80% of NCDs deaths occurred in low- and middle-income countries. As one of the most populated countries in the world, trends in Chinese residents’ mortality may serve as early markers to support worldwide progress in public health initiatives to improve life expectancy and quality of life.

In this study, we illustrate the mortality pattern changes and calculated the annual percentage changes (APC) in China during 2004–2016. In addition, we revealed and compared age-specific and disease-specific mortality rates of men and women in 2016. According to these findings, we could provide a scientific basis for the government to formulate policies and improve the health status of residents.

## Methods

### Data source

This analysis based on data of mortality from China Death Surveillance Database (2004–2016), which is compiled by National Health and Family Planning Commission of the People’s Republic of China, and is one of the most commonly used, highest-quality sources of detailed mortality. The database was collected by Death Surveillance Points system (DSPs), which was established with 2 surveillance points in 1978, increased to 145 points (placed in 31 provinces except for Hong Kong, Macao and Taiwan) and covered about 10 million population in1990. Subsequently, DSPs was expanded again in 2003 (included 161 points and covered about 77 million population). In 2013, DSPs covered about 24.30% population of China in 605 surveillance points. To ensure the representativeness, all the surveillance points were selected by utilizing an iterative method involving multistage stratification that considered the sociodemographic characteristics of the population [3]. The representativeness and quality of data collection in DSP has been validated in previous studies [4, 5]. Causes of death were categorized by Global Burden of Disease (GBD) and coded by International Classification of Diseases (ICD-10), which had a strict quality control over registration to ensure the authenticity and reliability of data. An additional file shows this in detail [see Additional file 1: Table S1].

### Statistical analysis

Age-standardized mortality rates (ASMR) for men and women during 2004–2016 were calculated according to the age distribution of the World Health Organization’s World Standard Population (2000–2025) [6]. We used log-linear model to check statistically significant trends in crude and age-standardized mortality rates, APC and measure the linear trend over the study period [7]. The chi-square statistical test was used to compare male and female death percentage in each specific age group (total 4 age groups) in 2016. The level of statistical significance was set at *P* < 0.05. All data analysis and figures were processed by R software, version 3.4.1 (R Foundation, Vienna, Austria).

## Results

### Mortality trends of Chinese men and women during 2004–2016

**Fig. 1:**
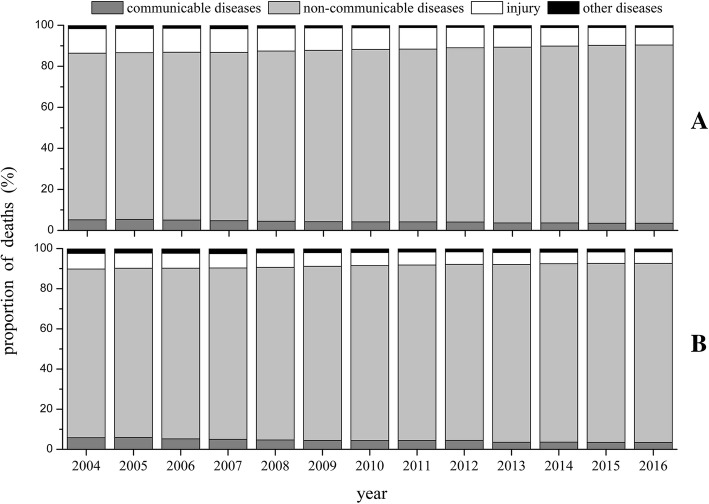
Comparisons of proportional trends of mortality pattern for men and women in China, 2004–2016. A: Proportion of death (%) for men. B: Proportion of death (%) for women. Cause of death definitions based on GBD Cause groups and ICD codes was showed in Additional file 1: Table S1

As shown in Table 1, annual crude mortality rates (CMR) and age-standardized mortality rates (ASMR) were higher in men than women between 2004 and 2016.The ASMR decreased significantly by 1.98% of APC for man and 2.45% for women over those 13 years. The comparisons of proportional trends of mortality pattern were illustrated in Fig. 1. The proportion of deaths of chronic non-communicable diseases in men and women had increased by 5.58 and 5.11% from 2004 to 2016. For infectious diseases, the proportion had declined by 1.63 and 2.31% in men and women, respectively. The proportion of deaths attributed to chronic non-communicable diseases were 86.81 and 89.13% in men and women in 2016, and the proportion due to infectious diseases were 3.55 and 3.47%.

**Table 1:** The mortality rates of Chinese men and women during 2004–2016

Years	CMR (per 100,000 per year)		ASMR (per 100,000 per year)	
	Men	Women	Men	Women
2004	687.88	519.29	801.19	529.36
2005	689.21	531.18	804.07	537.73
2006	601.90	446.29	690.89	441.25
2007	646.18	472.23	723.12	455.53
2008	665.09	479.82	717.58	454.44
2009	675.13	487.47	787.00	497.32
2010	667.73	479.25	802.41	504.19
2011	672.16	475.53	764.29	453.05
2012	601.12	465.33	654.48	398.68
2013	742.23	542.37	653.74	407.62
2014	746.29	545.68	640.02	398.01
2015	744.47	551.77	638.25	404.61
2016	750.68	562.92	607.47	379.96
APC(95%CI)(%)	1.09 (0.14,2.05)	1.00 (−0.01,2.02)	−1.98 (−2.93,-1.02)	−2.45 (−3.39,-1.50)
*P*-value	0.046	0.079	0.002	< 0.001

*CMR* Crude mortality rate, *ASMR* Age standardized mortality rate, *APC* Annual percentage change (%). *CI* Confidence interval

### Death status of Chinese residents in 2016

There were 1,743,541 deaths monitored by DSP system in China in 2016, and the crude mortality rate was 658.50 per 100,000 per year. The ASMR of total population were 490.28, 607.47 and 379.96 per 100,000 per year in men and women, respectively. Most of deaths occurred in men, accounting for 58.03% of the total deaths.

Disease-specific mortality rates based on different genders were shown in Table 2. The top five causes of deaths were malignant neoplasm, cerebrovascular disease (CVD), heart disease, Chronic Obstructive Pulmonary Disease (COPD) and accidental injury, accounting for 81.24 and 78.50% of deaths in men and women from all factors. The mortality rates of malignant neoplasm, CVD, COPD, accidental injury among men were higher than those of women.

**Table 2:** Disease-specific mortality rates of men and women in China in 2016

Disease	Deaths		% of total		ASMR (per 100,000 per year)		*χ* ^*2*^	*P*-value
	Men	Women	Men	Women	Men	Women		
malignant neoplasm	1,011,826	147,095	26.67	20.10	153.71	78.78	24.17	< 0.001
CVD	269,806	171,884	21.65	23.49	128.89	85.78	8.61	0.003
heart disease	219,011	153,991	16.61	21.05	101.42	76.55	3.24	0.072
COPD	168,110	67,658	8.99	9.25	55.30	33.12	5.50	0.019
accidental injury	90,974	33,732	7.32	4.61	48.09	20.29	11.53	0.001
hypertension	74,055	29,026	2.04	3.97	12.49	14.29	0.15	0.695
diabetes mellitus	20,597	20,900	1.80	2.86	10.57	10.70	0.00	1.000
LRTI	18,188	13,853	1.60	1.89	10.79	7.31	0.89	0.346
intentional injury	16,206	8207	1.17	1.12	7.29	4.84	0.33	0.564
cirrhosis	11,802	2959	0.99	0.40	5.70	1.56	1.13	0.289

*CMR*  = crude mortality rate. ASMR = age-standardized mortality rate. CVD = Cerebrovascular Disease. COPD = chronic obstructive pulmonary disease. LRTI = Lower Respiratory Tract Infections

**Fig. 2:**
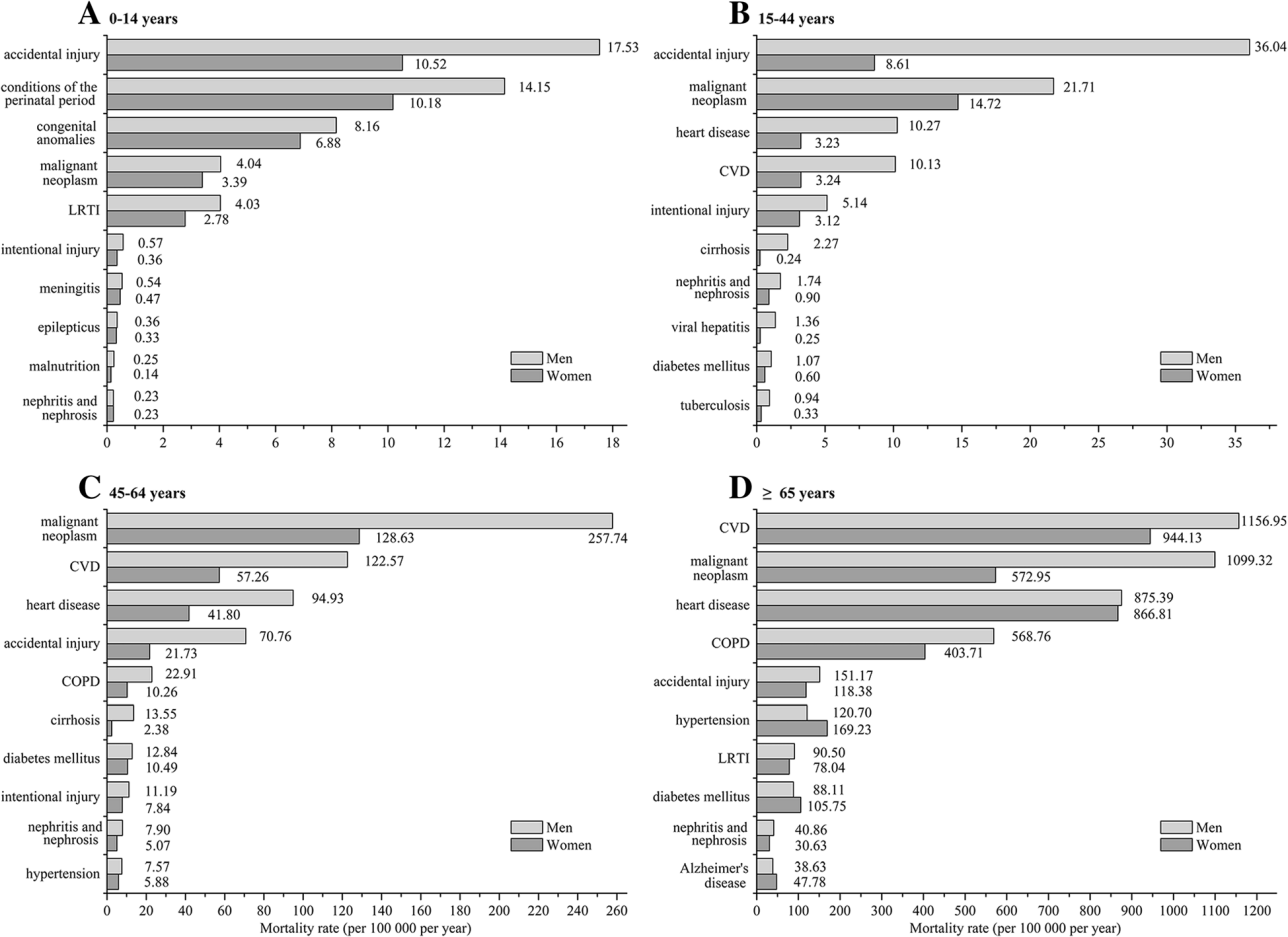
The mortality rates of 10 leading causes of death in Chinese population in 2016. The mortality rates (per 100,000 per year) of 10 leading causes of death were classified by sex and age group. A: 0–14 year old age group. B: 15–44 year old age group. C: 45–64 year old age group. D: ≥65 year old age group. COPD = chronic obstructive pulmonary disease

Male mortality rates were higher in all age groups than those in females (Table 3). The ASMR for the 10 leading causes of death among men and women were shown in Fig. 2. Age group 0–14 was the lowest mortality age group, and the main causes of death were accidental injury, conditions of the perinatal period and congenital anomalies. The top cause of death in men aged 15–44 years was accidental injury, and the mortality was 4.19 times than women in the same age group. Mortality rate of male aged 45–64 years with all causes of deaths was 2.16 times than female, especially cirrhosis (5.69 times). Malignant neoplasm, CVD, heart disease, accidental injury and COPD were the top five causes of death for men and women aged 45 years and above, and these mortality rates were higher in men. Mortality rates of hypertension, diabetes mellitus and Alzheimer’s disease of women aged 65 years and above were higher than men.

**Table 3:** Age-specific mortality rates of men and women in China in 2016

Age group	Deaths		% of total		CMR (per 100,000 per year)		*χ* ^*2*^	*P*-value
	Men	Women	Men	Women	Men	Women		
0–14	13,689	8423	1.35	1.15	59.47	42.57	588.76	< 0.001
15–44	63,034	24,691	6.23	3.37	106.08	43.04	15,450.10	< 0.001
45–64	261,139	118,014	25.81	16.13	687.87	318.29	50,966.59	< 0.001
≥ 65	673,964	580,587	66.61	79.35	4685.45	3684.22	18,899.28	< 0.001

CMR = crude mortality rate

## Discussion

Few studies have reported age-specific and cause-specific mortality in China. This study focused on mortality patterns change and death disparities among Chinese men and women. With improvements of disease control and residents’ health condition, the decline of mortality rates of Chinese population is obvious. Chronic non-communicable diseases were the most serious threat to the health of Chinese population in 2004–2016. The age-specific death pattern and mortality rate of men and women vary.

Historically, the main causes of death and disability in developing countries were infectious diseases and infant mortality [8]. In view of this situation, China has published laws to control infectious diseases and great achievements have been made [9]. Besides, to prevent the death of infants and young children, we should strengthen the perinatal health care and the screening of congenital anomalies. The results also indicated that prevention of deaths of malignant neoplasms and cardio-cerebrovascular diseases in 45 years and above have become the public health priorities in China.

According to Cancer Statistics in China in 2016, lung cancer, stomach cancer, liver cancer, esophageal cancer, and rectal cancer accounted the top five neoplasms for men, while lung cancer, stomach cancer, liver cancer, breast cancer, and esophageal cancer for women [10]. A great number of studies showed that air pollution and smoking are the main causes of lung cancer as well as chronic obstructive pulmonary disease. There were 316 million smokers in China, and the smoking rate reached a whopping 52.10% for men and 2.70% for women reported by Chinese center for disease control and prevention [11]. Lung cancer mortality rate was respectively 61.25 and 27.69 per 100,000 per year people for men and women. The following reasons explained this situation. Firstly, most Chinese women cook daily for their families, and specific cooking habits lead to indoor air pollution (using poorly ventilated coal stoves and kitchen fumes). Secondly, women’s exposure to passive smoking was extremely severe. Previous study showed that the number of non-smokers exposed to second-hand smoke was approximate 2.50 times of smokers [12]. The premature mortality rates in United States have a great decline, especially cancer death rate dropped 25% from 1991 to 2014 by tobacco control, disease screening and developing new therapies [13]. To reduce the mortality of malignant neoplasms effectively, China should also establish a systematic environmental protection system and implement large-scale tobacco control urgently. Fortunately, the government has devoted to following the Paris Agreement on climate change and the 2030 Agenda for Sustainable Development to provide a sustainable living environment [14].

The mortality rates of cerebrovascular diseases and cardiovascular diseases (mainly including heart diseases and hypertension) were on the rise especially in the twenty-first century [15]. In 2016, the mortality of cerebrovascular diseases was higher than that of malignant neoplasms in both men and women aged 65 years and above. As the study of Jiang He, hypertension was the leading preventable risk factor for death among Chinese adults [16]. However, the control rate of cardio-cerebrovascular diseases was low in Chinese population. Numerous studies indicated that dietary and life style were important factors to cardiovascular health [17, 18]. The INTERHEART China study showed that sodium rich foods as dietary patterns were positively associated with cardiovascular diseases [19]. The Chinese Longitudinal Healthy Longevity Survey (CLHLS) showed that frequency intake of fruit and vegetables were inversely associated with all-cause mortality and physical activity was beneficial for the prevention of premature death [20]. We suggest people increase the intake of vegetables, fruits and legume, reduce the intake of other foods and drinks, such as sugar sweetened beverages, red and processed meats, saturated and trans fat, refined cereals, sugar-rich desserts, and sodium rich foods.

Diabetes mellitus had become the 7th leading cause of death both in Chinese men and women in 2016, which was earlier than the prediction in WHO projects that diabetes would be the 7th leading cause of death in 2030 [21]. In addition, WHO estimated 1.6 million deaths were directly caused by diabetes in the world, and most patients lived in developing countries [22]. Thus, it is urgent for Chinese population to avoid or delay diabetes by healthy diet, physical activity, medication, regular screening and treatment for complications. Risk scores based on risk factors without invasive tests have been demonstrated as an effective and low cost tool for identifying the high-risk individuals of diabetes mellitus. A risk score model of diabetes mellitus had been developed according to the data of a nationwide study in China [23]. Risk score based on demographic, anthropometric, and clinical information without a laboratory test was a useful and cheap tool for a stepwise screening strategy for undiagnosed type 2 diabetes. This approach was cost effective in China.

Alzheimer’s disease was the 10th and 9th leading cause of death among men and women of 65 years old and above respectively. Moreover, with the aging population increasing, Alzheimer’s disease has become a serious family and social problem. It is necessary to strengthen the health management and improve the life quality of Alzheimer’s patients [24]. However, the etiology of Alzheimer’s disease is largely unclear, and there is no effective therapy for prevention or treatment. The search for common and rare genetic variants that contribute to Alzheimer’s disease risk has provided significant insights into the molecular pathways involved in Alzheimer’s disease pathogenesis and hinted at potential novel therapeutic targets. More than 30 loci have been implicated in Alzheimer’s disease by genome-wide association studies (GWAS) and whole genome/exome sequencing [25, 26].

Because injury was not given priority as a health problem in the early years, the corresponding control and prevention were not soundly developed [27]. In 2003, China launched a nationwide injury monitoring pilot program and optimized its management model in practice, working with US National Center for Injury Prevention and Control to reduce the damage caused to the masses [28]. The proportion of injury death has been gradually reduced since 2004. Accidental and intentional injuries were the major causes of death for young and middle-aged Chinese residents, with a much higher rate for men than women. Road injury and falls were the main contributors to accidental injury. Criminal law on drink driving has made some progress in China [28]. The increase in the number of automobiles and in traffic congestion has created a need for additional effective policies. Safety belts and helmets are the most practical way to reduce the risk of death in road injury. Exercise programs, rehabilitation, medication management, and treatment of vitamin D deficiency are the most efficient single interventions to prevent falls death [29]. Intentional injury violence was a complex phenomenon influenced by psychological and social perspective [30]. Self-harm and interpersonal violence were the major contributors to intentional injury. The first recommendation is to strengthen the policies addressing the social determinants of violence, such as education, poverty and economic inequality. The second recommendation is to ensure that existing laws for violence prevention are fully enforced. The third recommendation is to widely implement comprehensive services for victims of violence, for instance, to lessen psychological trauma.

The China Death Surveillance system provides data for people’s health, which is an important information source for the design of prevention strategies and policies. However, China still needs to learn from other countries to improve the survey methods and enrich the research content [31, 32]. For instance, we could expand the death surveillance points by setting up personal health record. Additionally, with the development of globalization, each nation should cooperate to fight against communicable diseases and non-communicable diseases. The prevention measures should be formulated to reduce disease burden and boost health development.

## Conclusions

In summary, there are severe health gaps and disparities between men and women, and the chronic non-communicable diseases continue to be a serious threat to the health of Chinese population. Disease prevention and control of China are facing dual challenges from non-communicable diseases and communicable diseases. To reduce diseases burden, strategies such as keeping healthy life style, environment protection, and diseases prevention and control should be adopted of no delay.

## Additional file

Additional file 1: **Table S1.** Cause of death definitions based on GBD Cause groups and ICD codes (DOCX 14 kb)

## Data Availability

The datasets analyzed during the current study are available in the National Data of China repository (http://data.stats.gov.cn/).

## Abbreviations

APC: Annual Percent Changes
ASMR: Age-standardized Mortality Rates
CMR: Crude Mortality Rates
COPD: Chronic Obstructive Pulmonary Disease
CVD: Cerebrovascular Disease
DSPs: Disease Surveillance Points system
GBD: Global Burden of Disease
ICD: International Classification of Diseases
LRTI: Lower Respiratory Tract Infections
NCDs: Non-communicable diseases
SDGs: Sustainable Development Goals
UNDP: United Nations Development Programme
WHO: World Health Organization
