# Supplementary material for: Mortality pattern trends and disparities among Chinese from 2004 to 2016
Source: BMC Public Health. 2019 Sep 2;19:780. doi: 10.1186/s12889-019-7163-9 (PMC6717976; doi:10.1186/s12889-019-7163-9)
Supplement: Supplementary file 1 — Table S1. Cause of death definitions based on GBD Cause groups and ICD codes (DOCX 14 kb) [file 12889_2019_7163_MOESM1_ESM.docx]

**Supplemental Table 1.** Cause of death definitions based on GBD Cause groups and ICD codes

| Disease | GBD Cause groups | ICD-10 Code |
| --- | --- | --- |
| Communicable diseases, maternal, prenatal and nutrition conditions | U001 | A00-B99, D50-D53, E00-E02, E40-E46, E50-E64, G00-G04, H65-H66, J00-J06, J10-J18, J20-J22, N70-N73, O00-O99, P00-P96 |
| tuberculosis | U003 | A15-A19, B90 |
| meningitis | U017 | A39, G00, G03 |
| viral hepatitis | U018, U019 | B16-B19 |
| lower respiratory infections | U039 | J10-J18, J20-J22 |
| conditions of the perinatal period | U049 | P00-P96 |
| Chronic non-communicable diseases | U059 | C00-C97, D00-D48, D54-D89, E03-E39, E65-E90, F00-F99, G05-G99, I00-I99, J23-J99, K00-K93, N00-N69, N74-N99, Q00-Q99, H01-H59, H60-H64, H67-H95, L00-L99, M00-M99 |
| malignant neoplasm | U060 | C00-C97 |
| malnutrition | U054, U057, U058 | D50-D53, D64.9, E00-E02, E40-E46, E50, E51-E64 |
| diabetes mellitus | U079 | E10-E14 |
| Alzheimers and other dementia | U087 | F01, F03, G30-G31 |
| epilepticus | U085 | G40-G41 |
| disease of heart | U105, U107, U109 | I01-I09, I20-I25, I30-I33, I38, I40, I42 |
| hypertension and complication | U106 | I10-I13 |
| cerebrovascular disease | U108 | I60-I69 |
| chronic obstructive pulmonary disease (COPD) | U112 | J40-J44 |
| cirrhosis | U117 | K70, K74 |
| nephritis and nephrosis | U121 | N00-N19 |
| congenital anomalies | U131 | Q00-Q99 |
| Injury | U148 | V01-V89 |
| accidental injury | U149 | V01-X59, Y40-Y86, Y88, Y89 |
| intentional injury | U156 | X60-Y09, Y35-Y36, Y87.0, Y87.1 |
| Other Cause of Death | -- | -- |
